# The role of tissue factor and autophagy in pulmonary vascular remodeling in a rat model for chronic thromboembolic pulmonary hypertension

**DOI:** 10.1186/s12931-016-0383-y

**Published:** 2016-05-27

**Authors:** Chaosheng Deng, Dawen Wu, Minxia Yang, Yunfei Chen, Haibo Ding, Zhanghua Zhong, Ningfang Lian, Qiaoxian Zhang, Shuang Wu, Kaixiong Liu

**Affiliations:** Division of Respiratory and Critical Care Medicine, First Affiliated Hospital of Fujian Medical University, Fuzhou, Fujian Province 350005 China

## Abstract

**Background:**

Few reports have examined tissue factor (TF) and autophagy expression in chronic pulmonary thromboembolic hypertension (CTEPH) animal models. *Objectives*: To investigate the role of tissue factor (TF), autophagy and their interactions during chronic thromboembolic pulmonary hypertension (CTEPH) pathogenesis in a rat model.

**Methods:**

Autologous blood clots were repeatedly injected into the left jugular vein of rats with injecting endogenous fibrinolysis inhibitor tranexamic acid (TXA). Mean pulmonary arterial pressure (mPAP), histopathology and TF, Beclin-1 and microtubule-associated protein 1 light chain (LC3) expression levels were detected.

**Results:**

The mPAP and vessel wall area/total area (WA/TA) ratio in the experiment group increased significantly (*P* < 0.05). TF mRNA and protein expression levels in the experiment group increased significantly (*P* < 0.05). Beclin-1 and LC3B mRNA and protein expression levels were lower in the experiment group (*P* < 0.05). The mPAP had a positive correlation with WA/TA ratio (*r* = 0.955, *P* < 0.05). Beclin-1 and LC3B protein expression had a negative correlation with the WA/TA ratio (*r* = -0.963, *P* < 0.05, *r* = -0.965, *P* < 0.05, respectively). TF protein expression had a negative correlation with both Beclin-1 and LC3B protein expression (*r* = -0.995, *P <*0.05, *r* = -0972, *P <* 0.05, respectively).

**Conclusions:**

A rat model of CTEPH can be established by repeatedly introducing autologous blood clots into the pulmonary artery with injecting TXA. TF and autophagy may play a key role during CTEPH pathogenesis, especially in vascular remodeling.

## Background

Pulmonary embolism (PE) refers to the obstruction of the pulmonary artery or one of its branches by an embolus (e.g., thrombus, tumor, air, or fat) originating elsewhere in the body. The most common pulmonary embolus is thrombus, also known as pulmonary thromboembolism (PTE). Chronic thromboembolic pulmonary hypertension (CTEPH) is regarded as a late sequela of PTE [1]. Several animal models of PTE have been developed to further elucidate the pathogenesis and pathophysiological changes of PTE [2]. A chronic pulmonary embolism (CPE) animal model can be induced by the injection of polidocanol foam into the peripheral veins of rabbits [3]. However, few reports have examined CTEPH animal models because of their robust fibrinolytic system. As in previous studies, we have successfully established a chronic PTE animal model for the study of lung ischemia reperfusion injury using tranexamic acid (TXA) that inhibits endogenous fibrinolysis [4]. Moreover, since large animals such as dogs are more expensive and prohibitive in terms of large-scale experiments, we have successfully established a rat model of CTEPH.

Thrombotic factor tissue factor (TF) is a membrane-bound protein initiation factor of the extrinsic coagulation pathway. The interaction between TF and factor VIIa (FVIIa) promotes the conversion of fibrinogen (Fg) into fibrin (Fb) [5]. A number of studies have shown that upregulated TF expression plays a critical role in the process of thrombosis [6, 7]. However, few reports have examined TF expression in an animal CTEPH model.

Autophagy is an evolutionarily conserved process for the turnover of misfolded or aggregated cytoplasmic proteins, damaged organelles, and intracellular pathogens utilizing a lysosome-dependent degradation pathway [8]. Beclin-1 is a key component of autophagy and microtubule-associated protein 1 light chain (LC3) is a good indicator of autophagy [9]; however, the extent of their involvement in the occurrence and progression of CTEPH vascular endothelial lesions through the regulation of autophagic capacity remains unclear [8].

Therefore, in this study, we examined TF, Beclin-1 and LC3 expression in the CTEPH rat model and determined their role and interactions during CTEPH thrombosis and remodeling of the pulmonary vasculature.

## Methods

### Animal preparation and grouping

All experimental and animal care protocols were approved by the animal ethics committee of the Fujian Medical University Institutional Animal Care (SYXK 2012-0001) and Use Committee and the Guide for the Care and Use of Laboratory Animals (NIH, Bethesda, MD, USA). Ninety 2–3 month old healthy male Sprague Dawley (SD) rats (weighing 250–300 g) were provided by the Experimental Animal Center of Fujian Medical University (China). The rats were randomly divided into a sham operation group (*n* = 45) and experimental group (*n* = 45). The experimental group rats received an additional injection of autologous blood clots 4 days following the first injection; the procedures in the sham operation group were the same as in the experimental group except that the rats were injected with 0.9 % NaCl instead of autologous blood clots. The rats were further divided into three subgroups according to the time of observation: 1-week subgroup (*n* = 15), 2-week subgroup (*n* = 15), and 4-week subgroup (*n* = 15). The animals were kept in an isolated location at 20–24 °C and 65–70 % humidity and their activity was restricted approximately one week prior to the operation in order to mimic blood stasis in the clinical practice. All rats had free access to food and water.

### Autologous blood clot preparation

Blood was collected from the orbital vein using a capillary glass tube (inner diameter, 1 mm) and placed in sterilized petri dishes at room temperature for 24 h. Clots were rush out with normal saline from capillary glass tubes and then trimmed to 3 mm lengths. After that, clots were aspirated into a syringe with 2 ml normal saline containing TXA (200 mg/kg/rat) and then flipped into a catheter connected to a 7 F needle for later use.

### Animal model induction

The rats were fixed to the operating table following anesthesia with an intraperitoneal injection of 10 % chloral hydrate (0.3 g/kg) and the left external jugular vein was subsequently separated under sterile conditions. Next, the previously prepared clots were injected into the left jugular vein of the rat. Rats in the sham operation group were administered with 2 ml of 0.9 % NaCl via the left jugular vein. Injections were repeated in both the experimental and sham operation group 4 days following the first injection. Endogenous fibrinolysis was inhibited during the experiment with an intraperitoneal injection of TXA (200 mg/kg, 1 time/day until rats were killed). After the injection, the animals were returned to their habitat. Enteric-coated indomethacin tablets (0.5 mg/kg, 3 times/ day for 3 days) were provided for relieving pain per oral. Prophylactic penicillin (10,000 U/kg/day, for 3 days) was also provided to prevent infection.

### Detection of pulmonary arterial pressure in rats

**Fig. 1:**
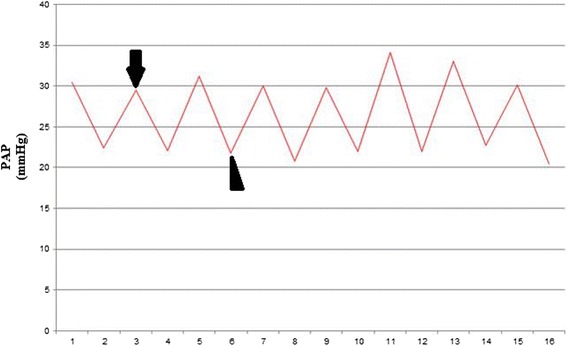
Detection of pulmonary arterial pressure in rats. Note: PAP = pulmonary arterial pressure. The curve waveform of pulmonary artery systolic pressure (*black arrow*) and diastolic pressure (*black arrow head*)

The rats were injected intraperitoneally with 10 % chloral hydrate (0.3 g/kg) and fixed in a supine position at either 1, 2, or 4 weeks following the second injection. The right external jugular vein was isolated and a PE-50 polyvinyl chloride (PVC) catheter connected to a pressure transducer and the biological signal acquisition system was slowly inserted. Catheter position was determined in accordance with changes in the pressure curve waveform and the catheter was extended into the superior vena cava, right atrium, right ventricle, and pulmonary arteries (Fig. 1).

### Histopathology

Lung tissues were selectively fixed with 10 % formaldehyde for 24 h, embedded in paraffin, and stained with hematoxylin and eosin (HE). The pathological changes in the pulmonary arteries were observed using an optical microscope (Leica DMI3000M, Germany) and the pulmonary arterial (50–100 μm) vessel wall area/total area (WA/TA) ratio was calculated using Images-Pro Plus (Mass precision instrument equipment co., LTD, Shenzhen, China) to evaluate the level of pulmonary arterial remodeling.

### Plasma TF concentrations

TF antigen concentration and activity in the plasma at 1, 2, and 4 weeks were determined using an enzyme-linked immunosorbent assay (ELISA) kit (Bluegene Biotech CO., LTD, Shanghai, China) and a Rat TF Chromogenic activity assay kit (Abcam (Shanghai) trading co., LTD, Shanghai, China), respectively, according to the manufacturer’s instructions.

### Immunohistochemistry

Rat pulmonary artery sections were deparaffinized and rehydrated using an alcohol gradient. Following blocking and antigen retrieval, sections were incubated with rabbit anti-rat TF, Beclin-1, and LC3B polyclonal antibodies (at 1:50, 1:150, 1:150, respectively; Abcam (Shanghai) trading co., LTD, Shanghai, China). Next, the sections were treated with the Biotin-Streptavidin Horseradish Peroxidase (HRP) Detection System (ZSGB-BIO, Shanghai, China) according to the manufacturer’s instructions. Additional lung specimens incubated with 1 % bovine serum albumin (BSA) served as the negative controls. Immunoreactivity was visualized using 3,3′- diaminobenzidine (DAB). The primary and secondary antibodies were omitted as a negative control for every group of slides.

### RT-PCR

RNA was extracted from the pulmonary artery tissue samples with the Trizol reagent (Invitrogen, Carlsbad, CA, USA) and the purity of the RNA was analyzed using spectrophotometer (MACY (China) INSTRUMENTS INC, Shanghai, China). Reverse transcription was performed in accordance with the manufacturer’s instructions of First Strand cDNA Synthesis Kit (Sangon Biotech Co., Ltd, Shanghai, China).

Primer pairs were designed for TF, Beclin-1, LC3B, and β-actin (internal control) using their DNA sequences (Sangon Biotech Co., Ltd, Shanghai, China). TF forward: 5′-AGA TGG AGG TGG AGA TGT GG-3′ and TF reverse: 5′-AAC AGC AGG TCT TTC CCA AG-3′; Beclin-1 forward 5′-TGT TTG GAG ATG TTG GAG CA-3′ and Beclin-1 reverse: 5′-ATG GAA GGT CGC ATT GAA GA-3′; LC3B forward: 5′-GTC TTT GTG GGT TGG ACC TC-3′ and LC3B reverse: 5′-TGG ATT TCT TCA GTT GCT TGG-3′; β-actin forward: 5′-AAC CCT AAG GCC AAC CGT G-3′ and β-actin reverse: 5′-TGC TCG AAG TCT AGG GCA AC-3′. Cycling conditions were as follows: 94 °C denaturation for 5 min; 35 cycles of 94 °C for 30 s, 60 °C for 30 s, and 72 °C for 30 s; and a final 10 min 72 °C extension. The PCR products were subjected to electrophoresis on a 2 % agarose gel containing 0.5 μg/ml ethidium bromide and the bands were visualized using a gel imaging system (HBMA-9600, China). TF, Beclin-1, and LC3B expression was semi-quantitatively determined with the imaging software.

### Western blot analysis

TF, Beclin-1, and LC3B protein expression was analyzed by western blot analysis. Protein concentrations were estimated using the Lowry protein assay. Proteins were separated by sodium dodecyl sulfate polyacrylamide gel electrophoresis (SDS-PAGE). Following blocking with BSA, the membranes were incubated overnight at 4 °C with anti-rat TF, anti-Beclin1 and anti-LC3 (1:500, 1:500, 1:1000, respectively; Abcam (Shanghai) trading co., LTD, Shanghai, China). The membranes were then incubated with HRP-conjugated secondary antibody (originated from rabbits) for 2 h at room temperature and the targeted antigens were detected using enhanced chemiluminescence reagents. The targeted proteins were analyzed using the Lab-work image analysis software (Gene Company Limited, Hong Kong, China).

### Statistical analysis

SPSS 17.0 (IBM, Armonk, NY, USA) software was used for statistical analyses. Numerical parameters with normal Gaussian distribution were expressed as means ± standard deviation (SD). Parameters measured at various time points within each group or subgroup were compared by variance analysis and the Pearson correlation coefficient was used to determine the correlation between two variables. *P* values <0.05 was considered as significant differences.

## Results

### Pulmonary arterial pressure in the rat model of CTEPH

The pulmonary arterial pressure in the experimental group and sham operation group were measured; the mean pulmonary arterial pressure (mPAP) increased gradually subsequent to repeated embolization in the 1-, 2-, and 4-week subgroups in the experimental group. The mPAP was significantly increased (*P* < 0.05) in the 4-week group compared with the 1-week group (Table 1).

**Table 1:** Mean pulmonary arterial pressure and vessel wall area/total area ratio in the rat CTEPH model (*x* ± SD)

Parameters	Subgroup (each *n* = 15)	Sham	Experimental group
mPAP (mmHg)			
	1-week	14.4 ± 2.66	18.97 ± 3.87^a^
	2-week	13.9 ± 2.82	21.89 ± 4.22^ad^
	4-week	14.1 ± 3.12	25.17 ± 4.74^ab^
WA/TA ratio			
	1-week	31.2 ± 6.25	46.67 ± 8.50^a^
	2-week	30.1 ± 6.01	49.06 ± 8.82^ad^
	4-week	32.8 ± 6.83	54.03 ± 10.26^ac^

*mPAP* mean pulmonary arterial pressure, *WA/TA* Vessel wall area/total area

^a^indicated that mPAP and WA/TA in the experimental group compared to sham operation group (*P* < 0.05, respectively) in the 1-, 2-, and 4-week subgroups.

^b^indicated that the mPAP in the 4-week subgroup compared to 1-week subgroup (*P* <0.05) and 2-week subgroup (*P >*0.05) in the experimental group.

^c^indicated that the WA/TA in the 4-week subgroup compared to 1-week subgroup (*P >*0.05) and 2-week subgroup (*P >*0.05) in the experimental group.

^d^indicated that the mPAP and WA/TA in the 2-week subgroup compared to 1-week subgroup (*P >*0.05, respectively) in the experimental group

### Pathological changes in the rat model of CTEPH

**Fig. 2:**
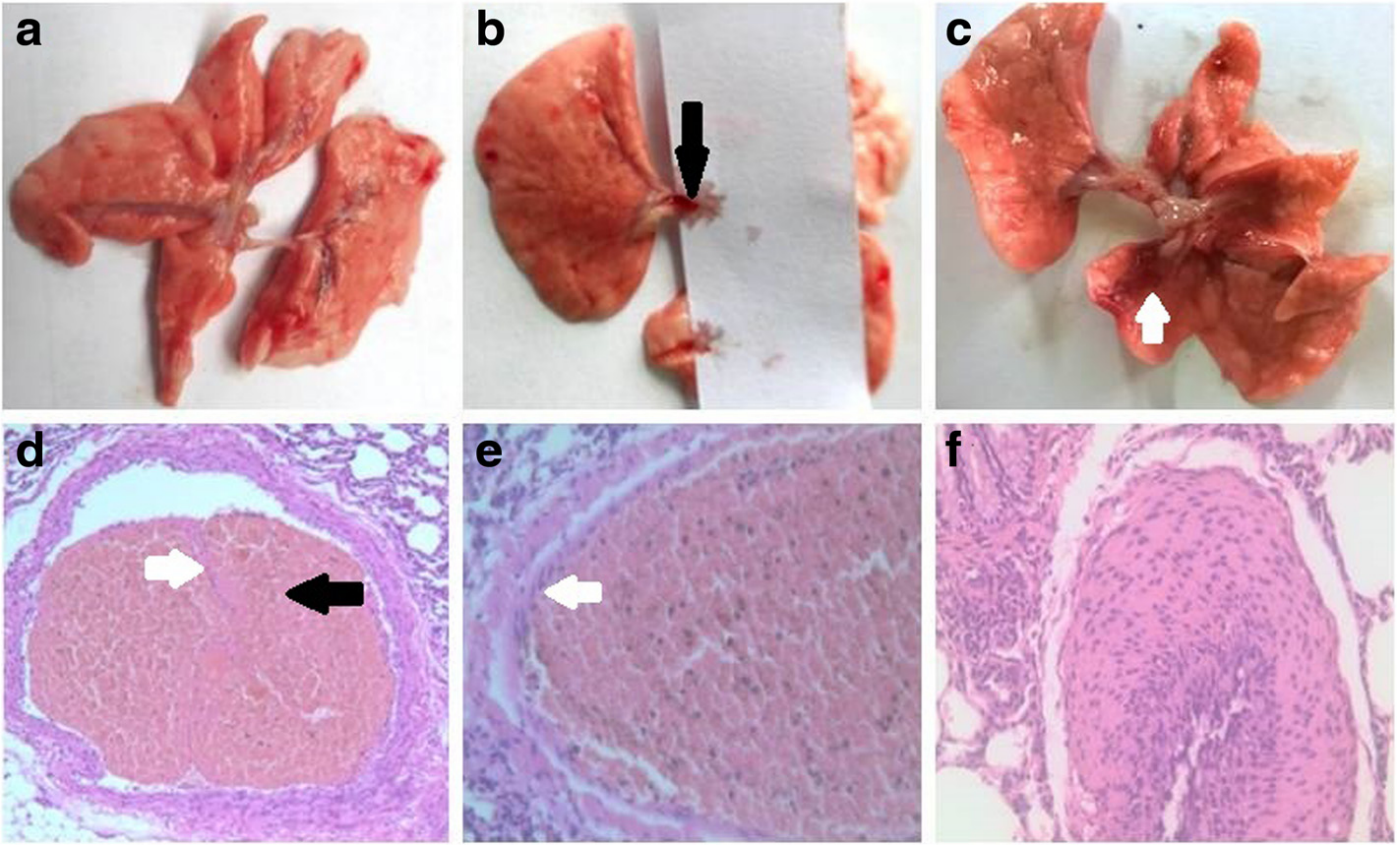
Pathological changes in the animal model of CTEPH. Note: No significant changes were apparent in the sham group (**a**). Macroscopic pathology demonstrated reddish-brown thrombi (*black arrow*) adhered to the left lower lobar artery wall in the 1-week and 2-week subgroups (**b**) and a dark red pulmonary infarction (*white arrow*) in the 4-week group (**c**) following repeated embolization. Histological sections of pulmonary artery demonstrated organized tissue (*white arrow*) on the surface of the thrombi (*black arrow*) and invasive growth into it (**d**, HE, 20×). The pulmonary artery endothelial cells (*white arrow*) were closely connected with the thrombus (**e**, HE, 40×) and the thickened endothelium was present in the distal pulmonary artery (**f**, HE, 40×)

No significant changes were apparent in the sham operation group (Fig. 2a). Macroscopic pathology demonstrated reddish-brown thrombi adhered to the left lower lobar artery wall in the 1-week and 2-week subgroups (Fig. 2b) and dark red pulmonary infarctions in the 4-week subgroup (Fig. 2c) in the experimental group following repeated embolization. Histological sections of the pulmonary artery demonstrated organized tissue covering the surface of the thrombus and invasive growth into it (Fig. 2d). The pulmonary artery endothelial cells were closely connected with the thrombus (Fig. 2e) in the thromboembolic pulmonary artery and a thickened intima was apparent in the distal pulmonary artery (Fig. 2f). As shown in Table 1, the WA/TA ratio in the 1-week, 2-week, and 4-week subgroups in the experimental group increased significantly (*P* < 0.05) compared to the sham operation group.

### TF antigen plasma concentration and activity

**Fig. 3:**
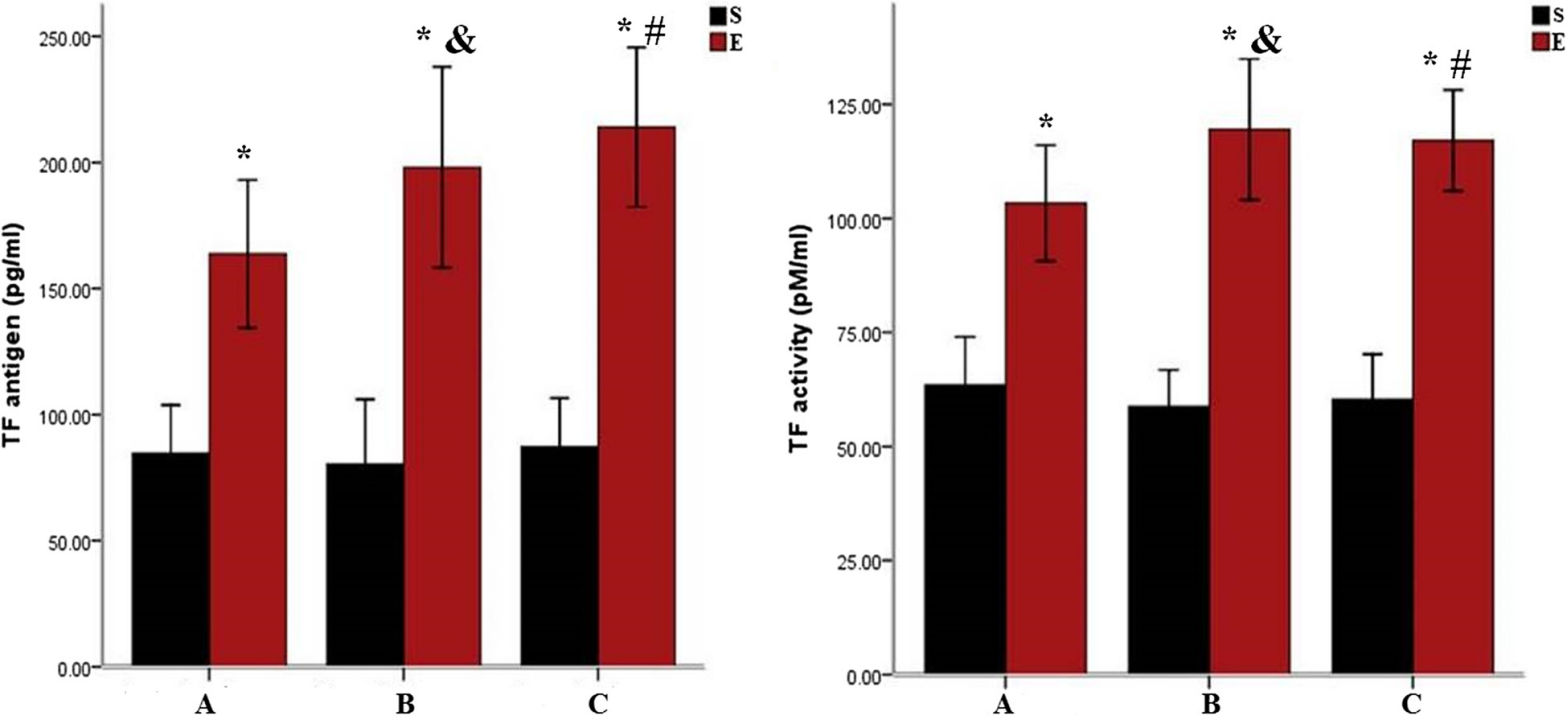
TF antigen expression in rat plasma from different groups. Note: S = sham operation group, E = experimental group. *a* 1-week subgroup; *b* 2-week subgroup; *c* 4-week subgroup. * indicated that TF antigen expression and activity in the plasma of experimental group compared to sham operation group (*P* < 0.05, respectively) in the 1-, 2-, and 4-week subgroups. ^#^ indicated that the TF antigen expression and activity in the plasma of 4-week subgroup compared to 1-week subgroup (*P >*0.05, respectively) and 2-week subgroup (*P >*0.05, respectively) in the experimental group. & indicated that the TF antigen expression and activity in the plasma of 2-week subgroup compared to 1-week subgroup (*P >*0.05, respectively) in the experimental group

The plasma TF concentration and activity from different groups were showed in Table 2. TF concentration in the 1-week, 2-week, and 4-week subgroups in the experimental group increased significantly compared with the sham operation group (*P* < 0.05, respectively). However, no significant differences were apparent between the 1-, 2-, and 4-week subgroups in the experimental group (Fig. 3A). Rat plasma TF activity was higher in the 1-, 2-, and 4-week subgroups in the experimental group than in the sham group (*P* < 0.05, respectively). However, there were no significant difference between the 1-, 2-, and 4-week subgroups in the experimental group (Fig. 3B).

**Table 2:** TF antigen expression and activity in rat plasma from different groups $\left(x\pm \mathrm{S}\mathrm{D}\right)$

Parameters	Subgroup (each *n* = 15)	Sham	Experimental group
TF antigen			
(pg/ml)	1-week	83.24 ± 21.90	170.98 ± 43.06^a^
	2-week	82.45 ± 22.76	205.50 ± 45.71^ac^
	4-week	83.92 ± 24.11	218.97 ± 35.68^ab^
TF activity			
(pM/ml)	1-week	62.62 ± 16.06	103.39 ± 22.05^a^
	2-week	60.15 ± 17.23	119.54 ± 26.70^ac^
	4-week	61.87 ± 16.69	117.12 ± 19.14^ab^

*TF* tissue factor

^a^indicated that TF antigen and activity in the experimental group compared to sham operation group (*P* < 0.05, respectively) in the 1-, 2-, and 4-week subgroups

^b^indicated that TF antigen and activity in the 4-week subgroup compared to 1-week subgroup (*P* >0.05, respectively) and compared to 2-week subgroup (*P* >0.05, respectively) in the experimental group

^c^indicated that TF antigen and activity in the 2-week subgroup compared to 1-week subgroup (*P* >0.05, respectively) in the experimental group

### TF, Beclin-1, and LC3B antigen expression in the pulmonary artery following repeated embolization

**Fig. 4:**
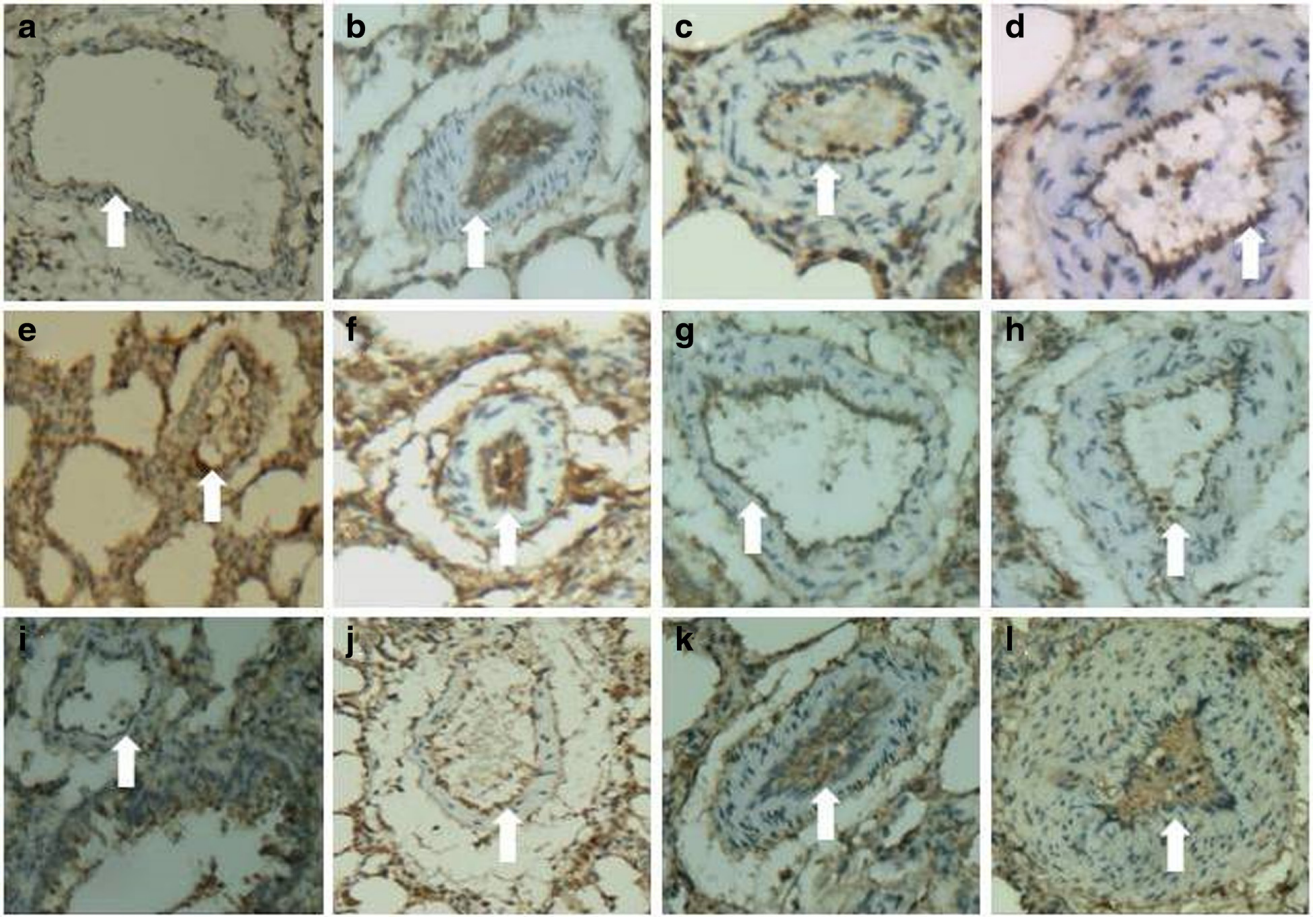
Immunohistochemical analysis of TF, Beclin-1 and LC3B antigen expression in the pulmonary artery. Note: TF antigen was expressed in the pulmonary artery intima of rats in both the sham operation and experimental group. TF antigen expression was low in the pulmonary arterial intima of the sham operation group (**a**). However, TF antigen expression was increased in the experimental group in 1-, 2-, and 4-week subgroups (**b**, **c**, **d**, respectively). Beclin-1 and LC3B antigen were expressed in the pulmonary artery of rats in both the sham operation and experimental groups. The expression of Beclin-1 (**f**, **g**, **h**) and LC3B (**j**, **k**, **l**) antigen in the experimental groups was lower than in the sham operation group (**e**, **i**), especially in the pulmonary arterial intima

The TF antigen was visualized with DAB and all brownish red staining were attributed to the TF antigen. In the sham operation group, TF antigen expression was concentrated mainly in the pulmonary artery adventitia and rarely in the intima (Fig. 4a). In contrast, in the experimental group, TF antigen expression was increased significantly in the intima (Fig. 4b-d). Beclin-1 (Fig. 4f-h) and LC3B (Fig. 4j-l) antigen expression was lower in the experimental group than in the sham operation group (Fig. 4e, i), especially in the pulmonary arterial intima.

### TF, Beclin-1, and LC3B mRNA expression levels in the pulmonary artery following repeated embolization

**Fig. 5:**
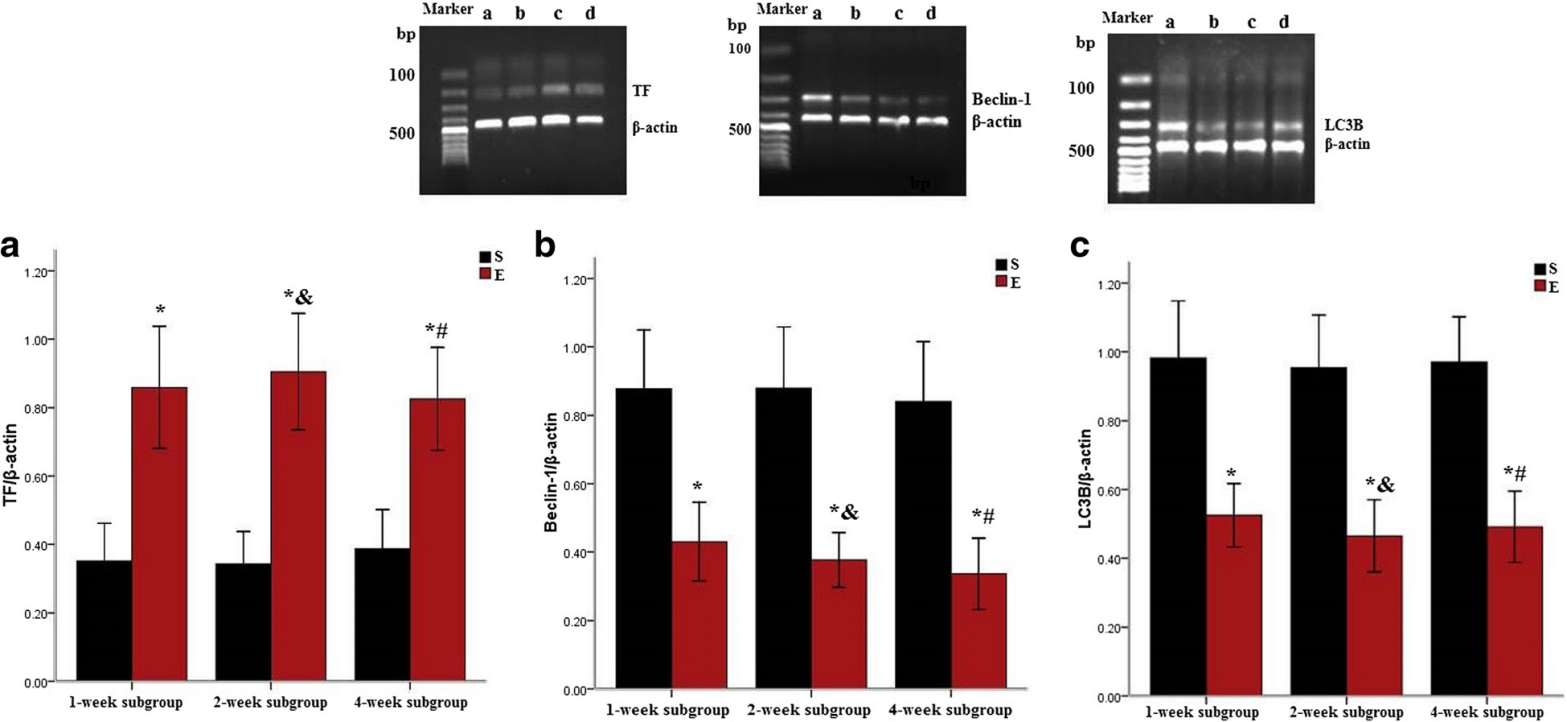
TF, Beclin-1, and LC3B mRNA expression in the pulmonary artery. Note: S = sham operation group, E = experimental group. *a* Sham operation group; *b* 1-week subgroup in experimental group; *c* 2-week subgroup in experimental group; *d* 4-week subgroup in experimental group. TF, Beclin-1, and LC3B mRNA were expressed in the pulmonary artery of rats in both the sham operation and experimental groups. TF mRNA expression in the experimental groups was increased compared to the sham operation group (**a**). In contrast, the Beclin-1 and LC3B mRNA expression was lower in the experimental groups than in the sham operation group (**b**, **c**). * indicated that TF, Beclin-1, and LC3B mRNA expression of experimental group compared to sham operation group (*P* < 0.05, respectively) in the 1-, 2-, and 4-week subgroups. ^#^ indicated that the TF, Beclin-1, and LC3B mRNA expression of 4-week subgroup compared to 1-week subgroup (*P >*0.05, respectively) and 2-week subgroup (*P >*0.05, respectively) in the experimental group. & indicated that the TF, Beclin-1, and LC3B mRNA expression of 2-week subgroup compared to 1-week subgroup (*P >*0.05, respectively) in the experimental group

TF expression was significantly increased (*P* < 0.05) in the 1-, 2-, and 4-week subgroups in the experimental group compared with the sham operation group. Beclin-1 and LC3B expression was lower (*P* < 0.05) in the 1-, 2-, and 4-week subgroups in the experimental group compared with the sham group, however, there were no significant differences between the three experimental subgroups (Table 3, Fig. 5).

**Table 3:** Tissue factor, Beclin-1, and microtubule-associated protein 1 light chain mRNA expression in the pulmonary artery (*x* ± SD)

Parameters	Subgroup (each *n* = 15)	Sham	Experimental group
TF/β-actin			
	1-week	0.35 ± 0.15	0.85 ± 0.23^a^
	2-week	0.39 ± 0.18	0.96 ± 0.26^ac^
	4-week	0.41 ± 0.21	0.84 ± 0.22^ab^
Beclin-1/β-actin			
	1-week	0.88 ± 0.31	0.43 ± 0.20^a^
	2-week	0.81 ± 0.28	0.38 ± 0.14^ac^
	4-week	0.94 ± 0.37	0.51 ± 0.29^ab^
LC3B/β-actin			
	1-week	0.98 ± 0.28	0.51 ± 0.19^a^
	2-week	0.89 ± 0.21	0.45 ± 0.15^ac^
	4-week	0.96 ± 0.24	0.49 ± 0.18^ab^

*TF* tissue factor, *LC3* microtubule-associated protein 1 light chain, *LC3B* a phosphatidylethanolamine conjugated form of LC3

^a^indicated that TF, Beclin-1 and LC3B in the experimental group compared to sham operation group (*P* < 0.05, respectively) in the 1-, 2-, and 4-week subgroups

^b^indicated that the TF, Beclin-1 and LC3B in the 4-week subgroup compared to 1-week subgroup (*P >*0.05, respectively) and compared to 2-week subgroup (*P >*0.05, respectively) in the experimental group

^c^indicated that the TF, Beclin-1 and LC3B in the 2-week subgroup compared to 1-week subgroup (*P* >0.05, respectively) in the experimental group

### TF, Beclin-1, and LC3B protein expression in the pulmonary artery following repeated embolization

**Fig. 6:**
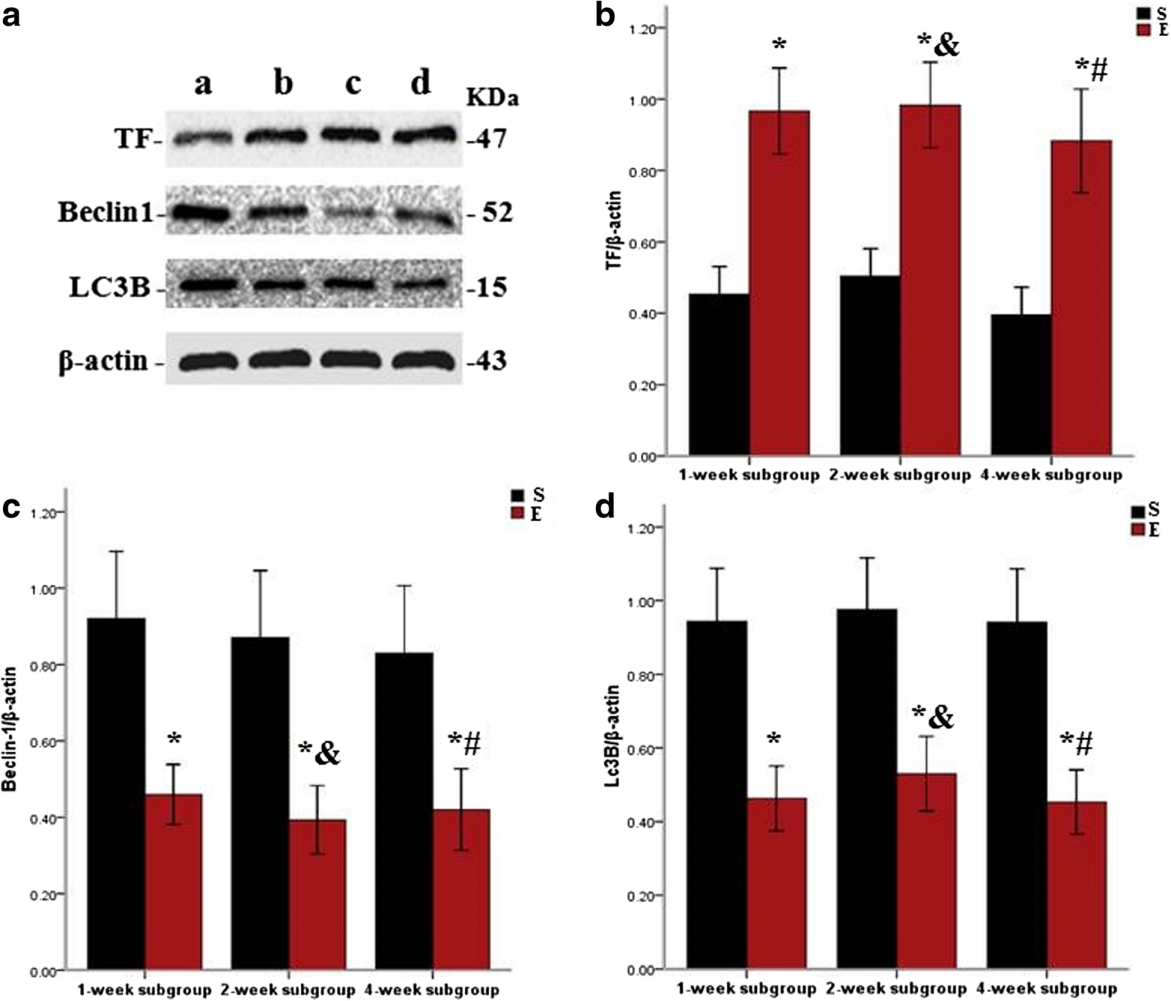
Western blot analysis of TF, Beclin-1, and LC3B protein expression in the pulmonary artery. Note: S = sham operation group, E = experimental group. *a* Sham operation group; *b* 1-week subgroup in experimental group; *c* 2-week subgroup in experimental group; d: 4-week subgroup in experimental group. TF, Beclin-1, and LC3B protein were expressed in the pulmonary artery of rats in both the sham operation and experimental groups (**a**). TF protein expression in the experimental groups was increased compared to the sham operation group (**b**). In contrast, the Beclin-1 and LC3B protein expression was lower in the experimental groups than in the sham operation group (**c**, **d**). * indicated that TF, Beclin-1, and LC3B protein expression of experimental group compared to sham operation group (*P* < 0.05, respectively) in the 1-, 2-, and 4-week subgroups. ^#^ indicated that the TF, Beclin-1, and LC3B protein expression of 4-week subgroup compared to 1-week subgroup (*P >*0.05, respectively) and 2-week subgroup (*P >*0.05, respectively) in the experimental group. & indicated that the TF, Beclin-1, and LC3B protein expression of 2-week subgroup compared to 1-week subgroup (*P >*0.05, respectively) in the experimental group

As shown in Fig. 6A, TF, Beclin-1, and LC3B were expressed in the pulmonary artery of rats in the sham operation group (a), 1-week subgroup in experimental group (b), 2-week subgroup in experimental group (c), and 4-week subgroup in experimental group (d). TF protein expression in the pulmonary artery was significantly increased (*P* < 0.05) in the 1-, 2-, and 4-week subgroups in the experimental group. However, expression was not significantly different between the 1-, 2-, and 4-week subgroups (Fig. 6B). No significant difference were apparent comparing the expression of Beclin-1 and LC3B in the 1-, 2-, and 4-week subgroups in the experimental group; however, the expression of both proteins was reduced (*P* < 0.05) (Fig. 6C, D).

### Pearson correlation coefficient (r) for WA/TA ratio, mPAP, TF activity, and TF, Beclin-1, and LC3B protein expression

As shown in Table 4, the mPAP had a positive correlation with WA/TA ratio (*r* = 0.955, *P* < 0.05). TF activity in the plasma of rats had a positive correlation with the WA/TA ratio (*r* = 0.972, *P* < 0.05). Beclin-1 and LC3B protein expression had a negative correlation with the WA/TA ratio (*r* = -0.963, *P* < 0.05, *r* = -0.965, *P* < 0.05, respectively).

**Table 4:** Pearson correlation coefficient (r) for the WA/TA ratio, mPAP, TF activity and TF, Beclin-1, and LC3 protein expression in the pulmonary artery

	WA/TA ratio	mPAP	TF protein
mPAP	0.955 (*p* = 0.045)	–	–
TF activity	0.972 (*p* = 0.028)	0.914 (*p* = 0.086)	–
TF protein	0.894 (*p* = 0.110)	0.738 (*p* = 0.260)	–
Beclin-1 protein	−0.963 (*p* = 0.037)	−0.862 (*p* = 0.140)	−0. 995 (*p* = 0.005)
LC3B protein	−0.965 (*p* = 0.035)	−0.847 (*p* = 0.150)	−0.972 (*p* = 0.028)

*mPAP* mean pulmonary arterial pressure, *WA/TA* vessel wall area/total area, *TF* tissue factor, *LC3* microtubule-associated protein 1 light chain, *LC3B* a phosphatidylethanolamine conjugated form of LC3

### Pearson correlations coefficient (*r*) for TF, Beclin-1, and LC3B protein expression in the pulmonary artery

TF protein expression in the pulmonary artery had a negative correlation with both Beclin-1 and LC3B protein expression (*r* = -0.995, *P <*0.05, *r* = -0972, *P <* 0.05, respectively; Table 4).

## Discussion

### A rat model of CTEPH

Previously approved PTE animal models have been useful for understanding the pathogenesis and pathophysiological changes of PTE, evaluating methods for diagnosis, and providing new therapeutic approaches [2]. However, since the pathogenesis and pathological changes of CTEPH are complex, it is difficult to mimic the pathophysiological changes in an animal model [10]. Since the 1990s, several attempts to develop CTEPH animal models using microspheres or polidocanol foam have failed because of the high fibrinolysis potential of pulmonary endothelial cells and the adaptive capabilities of the pulmonary circulation [3, 11, 12]. However, an animal model of PTE has been developed using the injection of an exogenous thrombus from the femoral vein into the pulmonary artery to mimic the pathophysiology of deep vein thrombosis (DVT)-PTE [13]. Li et al. [14] have established a rat model of CTEPH by injecting thrombi twice in 2 weeks through the jugular vein.

In our study, we found that the mPAP gradually increased in the 1-, 2-, and 4-week subgroups in the experimental group, indicating that we have successfully established a rat model of CTEPH by repeated injection of autologous blood clots into the pulmonary artery. Our preliminary experiments demonstrated almost complete fibrinolysis of autologous clot PTE within 5 days, similar to the results for the minimally-invasive autologous clot PTE model developed in Sprague–Dawley and Copenhagen rats by Runyon et al. [15]. In order to extend the persistence time of autologous blood clots in animals and trigger the vasculopathy in the pulmonary artery, we repeated the injection of autologous clots into the pulmonary artery after 4 days. Concomitantly, because of the robust fibrinolytic system in the rats, TXA was injected in order to inhibit endogenous fibrinolysis in the animals [4, 15]. The positions of the rats were changed so that more blood clots could be injected into the pulmonary artery, for example, from supine position to prone position. The risk factors for DVT, which include hypercoagulability, vessel wall injury, and blood stasis, were proposed by Virchow over 150 years ago [16]. Therefore, we also restrained rat activity prior to- and post operation to mimic the pathophysiological changes of blood stasis. In this study, we have demonstrated that reddish-brown thrombi adhere to the lower lobar artery wall, the presence of tissue on the surface of the thrombi, invasive growth into the thrombi, pulmonary artery endothelial cells (PAECs) are closely connected with the thrombus in the pulmonary artery, and that the thickened intima is present in the distal pulmonary artery. These findings concur with our previous study showing that pathology is altered following PTE [17]. In addition, the WA/TA ratio gradually increased in the present study and there was a positive correlation between the WA/TA ratio and the mPAP. Therefore, the development of chronic pulmonary hypertension is associated with significant vascular remodeling due to the extensively thickened intima in the distal pulmonary artery caused by repeated embolization in the proximal pulmonary artery.

### Expression of TF and autophagy in the CTEPH model

#### TF expression in rats following repeated embolization

TF is a transmembrane glycoprotein that plays an essential role in triggering blood coagulation [18]. TF can be produced in endothelial cells and monocytes, however, blood mononuclear cells and endothelial cells do not express TF under normal circumstances [19–21]. Experimental studies in mice have revealed that circulating mononuclear phagocytes carrying TF contribute to thrombus formation [22]. In PTE patients, the plasma levels of TF produced by leukocytes were increased and played an important role in the pathogenesis of PTE [18]. A previous study has shown that monocyte and plasma TF levels were significantly increased in subjects with DVT compared to controls and monocyte TF levels correlated with plasma TF levels [23]. Therefore, in this model, the increased plasma TF following repeated embolization may be produced by monocytes. In addition, a previous study has shown that TF activity levels were elevated in patients with symptoms of PE [22].

In our study, plasma TF concentration and activity in the 1-week, 2-week, and 4-week subgroups in the experimental group increased significantly compared with the sham operation group, which may contribute to secondary thrombosis. In addition, there was a positive correlation between TF activity and WA/TA ratio. Therefore, increased TF activity may lead to gradual degenerative changes in pathology and increased mPAP following repeated embolization.

Under normal conditions, TF antigen expression was concentrated mainly in the pulmonary artery adventitia and rarely in the intima. Vessel wall TF expression can be elevated when intact endothelial cells are impaired; TF expression may originate from subendothelial cells exposed at sites of denuding injury and leukocytes adherent to the vessel wall [24]. Our immunohistochemistry results showed that the expression of TF antigen is increased in the intima. Concomitantly, the expression of TF mRNA and protein were significantly elevated in the pulmonary artery of the 1-, 2-, and 4-week subgroups in the experimental group, similar to the results of a previous study demonstrating that TF expression is significantly increased in a rabbit acute pulmonary embolism model [7]. In summary, we have demonstrated that the increase in TF expression may result in the formation and maintenance of thrombi. However, there were no significant differences between the 1-, 2-, and 4-week subgroups in the experimental group. It is possible that TF expression reach its maximal level due to leukocytes in the injured endothelial cells.

Therefore, TF may play an important role in the development of PTE and CTEPH, especially by contributing to vascular remodeling and PH.

#### Beclin-1 and LC3B expression in the pulmonary artery following repeated embolization

The proteins Beclin-1 and LC3B are recruited to autophagosomal membranes during the formation of autophagosomes. Once the autophagosomes fuse with lysosomes to form autophagolysosomes, Beclin-1 and LC3B present on the inner autophagosomal membrane are degraded by the lysosomal proteases [25]. Subsequent studies identified LC3 and Beclin-1 as essential markers for autophagy [26]. Impaired autophagic flux is characterized by accumulated autophagosomes and reduced formation of autophagolysosomes [27]. Therefore, the decreased Beclin-1 and LC3B protein levels indicate that more Beclin-1 and LC3B protein were degraded because of more autophagolysosomes were being formed or less autophagosomes were being formed due to impaired autophagic flux. Our results showed that the expression of Beclin-1 and LC3B were much lower in the pulmonary artery of the 1-, 2-, and 4-week subgroups in the experimental group than that in the sham group, especially in the pulmonary arterial intima. These findings suggest that there may be a defective autophagic process in the pulmonary arterial intima in the rat model of CTEPH.

Nguyen et al. [28] found that deletion of Beclin-1 reduced the plasminogen- induced autophagy and accelerated apoptosis, indicating that interruption of autophagy may give rise to an antiangiogenic effect on the endothelial cells. Moreover, Beclin-1 may form complexes with apoptotic protein Bcl-2 and thus lack of Beclin-1 may accelerate caspase dependent apoptosis [29]. In contrast, Lee et al. showed that expression of Beclin-1 in endothelial cells of hemizygous mice resulted in increased proliferation, migration, and tube formation compared to the wild-type cells [30]. These results suggest that inhibition of autophagy may promote a switch to apoptosis. Many researchers believe that apoptosis-resistance may result from an initial wave of endothelial cell apoptosis triggered by environmental stress that leads to hyperproliferative endothelial cells [31]. Therefore, the reduced expression of Beclin-1 demonstrated in our study may promote a switch to apoptosis, leading to apoptosis-resistant and hyperproliferative endothelial cells. A previous study has shown that deletion of LC3B increases the stabilization of hypoxia-inducible factor-a and the production of reactive oxygen species and contributes to endothelial cell proliferation [32]. It is possible that the autophagic protein LC3B exerts a protective function during the pathogenesis of CTEPH through the regulation of endothelial cell proliferation.

Therefore, defective autophagic processes in the pulmonary artery intima may result in decreased expression of Beclin-1 and LC3B and ultimately lead to PAEC proliferation in this CTEPH model. Previous findings support the hypothesis that autophagy plays a critical role in vascular remodeling by regulating smooth muscle cell phenotype transitions [33]. In the present study, Beclin-1 protein expression in the pulmonary artery had a negative correlation with the WA/TA ratio. LC3B protein expression also had a negative correlation with the WA/TA ratio. These findings suggest there may be a closed link between autophagy and pulmonary vascular remodeling in CTEPH.

### Correlation between TF, Beclin-1, and LC3B expression in the pulmonary artery

Under a number of pathological conditions, TF can upregulate the expression of vascular endothelial growth factor through the mitogen-activated protein kinase (MAPK) signal transduction pathway and thus promote vascular remodeling including endothelial cell proliferation and angiogenesis [34]. A recent study found that forkhead box transcription factor O-1 (FoxO1), a downstream signal of MAPK, could participate in regulating the endothelial cell autophagy [35]. Therefore, TF may regulate changes in autophagy capacity by transforming the activity of the autophagic upstream MAPK signal. Our study showed that TF protein expression in the pulmonary artery had a negative correlation with Beclin-1 and LC3B expression. Increased TF expression may reduce the level of autophagic capacity by regulating the activity of MAPK. Further studies are required to elucidate the underlying molecular mechanism.

### Study limitations

Although the process of CTEPH was successfully mimicked in this study, some flaws distinguish between our model and clinical CTEPH patients. While mPAP gradually increased in our model following repeated embolization, the severity of elevated pulmonary artery pressure was notably different from that of CTEPH patients. Thrombolysis was still apparent in our model; other procedures such as heating the autologous blood clots in a 70 °C water bath for 10 min prior to injection may be required to inhibit thrombi lysis [36]. The intima of rat pulmonary artery was difficult to separate and the further research will be mainly concentrated in the intima in our model to imitate the research of endarterectomized tissues from patients with CTEPH. In addition, elucidation of the complex nature of the mechanisms of vascular remodeling in clinical cases with CTEPH require further study.

## Conclusion

A rat model of CTEPH can be established by repeatedly introducing autologous blood clots into the pulmonary artery with injecting TXA. The WA/TA ratio and mPAP were significantly increased in this rat CTEPH model. TF expression was increased and Beclin-1 and LC3B expression was decreased in this rat CTEPH model. TF and autophagy may play a key role during CTEPH pathogenesis, especially in vascular remodeling. However, the detailed mechanisms underlying these processes warrant further investigation.

## Abbreviations

CPE, Chronic pulmonary embolism; CTEPH, Chronic pulmonary thromboembolic hypertension; DAB, 3,3′-diaminobenzidine; DVT, Deep vein thrombosis; HE, Hematoxylin and eosin; LC3, Microtubule-associated protein 1 light chain; MAPK, Mitogen-activated protein kinase; mPAP, Mean pulmonary arterial pressure; PAECs, Pulmonary artery endothelial cells; PE, Pulmonary embolism; PTE, Pulmonary thromboembolism; PVC, Polyvinyl chloride catheter; TF, Tissue factor; TXA, Tranexamic acid; WA/TA, Vessel wall area/total area; 
